# Postsynaptic GABA(B) Receptors Contribute to the Termination of Giant Depolarizing Potentials in CA3 Neonatal Rat Hippocampus

**DOI:** 10.3389/fncel.2017.00179

**Published:** 2017-06-28

**Authors:** Ilgam Khalilov, Marat Minlebaev, Marat Mukhtarov, Elvira Juzekaeva, Roustem Khazipov

**Affiliations:** ^1^INMED-INSERM, Aix-Marseille UniversityMarseille, France; ^2^Laboratory of Neurobiology, Kazan Federal UniversityKazan, Russia

**Keywords:** GABA, giant depolarizing potentials, neonatal, hippocampus, patch-clamp techniques, development

## Abstract

During development, hippocampal CA3 network generates recurrent population bursts, so-called Giant Depolarizing Potentials (GDPs). GDPs are characterized by synchronous depolarization and firing of CA3 pyramidal cells followed by afterhyperpolarization (GDP-AHP). Here, we explored the properties of GDP-AHP in CA3 pyramidal cells using gramicidin perforated patch clamp recordings from neonatal rat hippocampal slices. We found that GDP-AHP occurs independently of whether CA3 pyramidal cells fire action potentials (APs) or remain silent during GDPs. However, the amplitude of GDP-AHP increased with the number of APs the cells fired during GDPs. The reversal potential of the GDP-AHP was close to the potassium equilibrium potential. During voltage-clamp recordings, current-voltage relationships of the postsynaptic currents activated during GDP-AHP were characterized by reversal near the potassium equilibrium potential and inward rectification, similar to the responses evoked by the GABA(B) receptor agonists. Finally, the GABA(B) receptor antagonist CGP55845 strongly reduced GDP-AHP and prolonged GDPs, eventually transforming them to the interictal and ictal-like discharges. Together, our findings suggest that the GDP-AHP involves two mechanisms: (i) postsynaptic GABA(B) receptor activated potassium currents, which are activated independently on whether the cell fires or not during GDPs; and (ii) activity-dependent, likely calcium activated potassium currents, whose contribution to the GDP-AHP is dependent on the amount of firing during GDPs. We propose that these two complementary inhibitory postsynaptic mechanisms cooperate in the termination of GDP.

## Introduction

Giant Depolarizing Potentials (GDPs) are a ubiquitous network activity pattern observed both in neonatal rodent and fetal primate hippocampal slices and in the isolated intact rat hippocampus (Ben-Ari et al., 1989; Khalilov et al., 1997; Leinekugel et al., 1998; Khazipov et al., 2001; for reviews, Ben-Ari et al., 2007; Blankenship and Feller, 2010; Cherubini et al., 2011). GDPs are generated in the CA3 hippocampal region from where they propagate to CA1, dentate gyrus, septum and enthorhinal cortex, and underlie synchronous neuronal calcium oscillations and support plasticity in the developing hippocampal networks (Leinekugel et al., 1997; Garaschuk et al., 1998; Menendez de la Prida et al., 1998; Khalilov et al., 2003; Gaïarsa, 2004; Kasyanov et al., 2004; Mohajerani and Cherubini, 2006; Wester and McBain, 2016). At the cellular level, GDPs are characterized by quasi-periodic synchronous depolarization and firing of CA3 pyramidal cells and interneurons followed by rebound afterhyperpolarization (AHP). Membrane potential dynamics in CA3 pyramidal cells during depolarization phase of GDPs is determined by the two principal mechanisms: (i) synaptic connections, including recurrent glutamatergic connections between CA3 pyramidal cells and GABAergic inputs, which transiently change their actions from depolarizing at the GDPs’ onset to the hyperpolarizing at the GDPs’ peak (Ben-Ari et al., 1989; Leinekugel et al., 1997; Bolea et al., 1999; Khalilov et al., 2015); and (ii) subthreshold voltage-gated conductances supporting intrinsic bursting of CA3 pyramidal cells (Sipila et al., 2006). The mechanisms underlying rebound hyperpolarization curtailing GDPs (hereafter referred to as GDP-AHP) remain less well understood, however.

Previous studies using whole-cell recordings from CA3 pyramidal cells suggested that GDP-AHP involves calcium-activated potassium channels which are activated by transient elevation of intracellular calcium concentration evoked by bursts of action potentials (APs) in CA3 pyramidal cells during GDPs (Sipila et al., 2006). Activation of postsynaptic GABA(B) receptor activated potassium currents is yet another possible mechanism involved in GDP-AHP. While neonatal CA3 pyramidal cells and neocortical neurons lack late GABA(B) receptor mediated component during monosynaptically evoked GABAergic responses, they start to respond to the GABA(B) receptor agonist baclofen as early as at the postnatal day P3 (Luhmann and Prince, 1991; Fukuda et al., 1993; Gaiarsa et al., 1995; Caillard et al., 1998; Nurse and Lacaille, 1999; Verheugen et al., 1999). This raises a possibility that postsynaptic GABA(B) receptors could be activated by massive release of GABA during GDPs. Moreover, blockade of GABA(B) receptors was shown to prolong GDPs and transform them to the interictal- and ictal-like discharges (McLean et al., 1996). These effects may involve inhibitory GABA actions on GABA(B) receptors located pre- and postsynaptically. However, analysis of the effect of the GABA(B) receptor antagonists on GDPs has been only performed using potassium channel blockers in the pipette solution (McLean et al., 1996) and the contribution of the postsynaptic GABA(B) receptors to the GDP-AHP remains elusive.

In the present study, we performed simultaneous gramicidin perforated patch-clamp and local field potential (LFP) recordings in order to study the mechanisms underlying GDP-AHP in CA3 pyramidal cells. We provide evidence that GDP-AHP involves two different yet complementary mechanisms: (i) postsynaptic GABA(B) receptor activated potassium currents, which are activated independently of whether cells fire or not during GDPs; and (ii) an activity-dependent, likely calcium activated potassium currents, whose contribution to the GDP-AHP depends on the amount of firing during GDPs. Cooperation of these two postsynaptic mechanisms supports feedback network and cellular control over GDPs.

## Materials and Methods

### Ethical Approval

All animal-use protocols followed the guidelines of the French National Institute of Health and Medical Research (INSERM, protocol N007.08.01) and the Kazan Federal University on the use of laboratory animals (ethical approval by the Institutional Animal Care and Use Committee of Kazan State Medical University N9-2013).

### Brain Slice Preparation

Horizontal brain slices were prepared from P4 to P8 Wistar rats. P0 was the day of birth. The animals were decapitated and the brain was rapidly removed and placed into oxygenated (95% O_2_–5% CO_2_) ice-cold (2–5°C) artificial cerebrospinal fluid (ACSF) of the following composition (in mM): NaCl 126, KCl 3.5, CaCl_2_ 2, MgCl_2_ 1.3, NaHCO_3_ 25, NaH_2_PO_4_ 1.2 and glucose 11 (pH 7.4). Four to five hundred micrometer thick horizontal slices were cut using a Vibratome (VT 1000E; Leica, Nussloch, Germany). Slices were kept in oxygenated ACSF at room temperature (20–22°C) for at least 1 h before use. For recordings, slices were placed into a submerged chamber and superfused with oxygenated ACSF at 30–32°C at a flow rate of 2–4 ml/min.

### Electrophysiological Recordings

Extracellular recordings of the LFPs and multiple unit activity (MUA) were performed from the pyramidal cell layer of CA3 region using extracellular 50 μm tungsten electrodes (California Fine Wire, Grover Beach, CA, USA) or with ACSF filled glass electrodes (2–3 MΩ). The signals from extracellular recordings were amplified and filtered (10,000×; 0.1 Hz–10 kHz) using a DAM80i amplifier, digitized at 10 kHz and saved on a PC for *post hoc* analysis.

Visual patch-clamp recordings were performed from CA3 pyramidal cells located in the vicinity of the extracellular electrode (separation distance <100 μm) using Axopatch 200B or MultiClamp700B (Axon Instruments, Union City, CA, USA) amplifiers. Patch electrodes were made from borosilicate glass capillaries (GC150F-15, Clark Electromedical Instruments) and had a resistance of 4–7 MΩ. The patch pipette solution for gramicidin perforated patch recording contained (in mM): KCl 150 and HEPES 10, buffered to pH 7.2 with Tris-OH. Gramicidin was first dissolved in DMSO to prepare a stock solution of 40 mg/ml and then diluted to a final concentration of 80 μg/ml in the pipette solution. The gramicidin containing solution was prepared and sonicated <1 h before the experiment. To facilitate cell-attached formation (4–10 GΩ) the tip of pipette was filled with a gramicidin-free solution. At 20–30 min after cell-attached formation, series resistance (*Rs*) decreased and stabilized at 12–61 MΩ. Whole cell recordings were performed using pipette solutions of the following composition (mM): 130 Potassium methanesulfonate; 1 CaCl_2_; 10 HEPES; 10 EGTA; pH 7.3. Series resistance was monitored for the duration of the recording session. A picospritzer (General Valve Corporation, Fairfield, NJ, USA) was used to puff-apply baclofen (300 μM in ACSF) or GABA (100 μM in ACSF, in the presence of the ionotropic GABA(A) and glutamate receptor antagonists) from a glass pipette in *stratum radiatum* at a distance of about 100 μm from the soma. The pressure varied from 10 psi to 20 psi, and the duration of the puff was 100 ms. In a set of experiments describing the effects of the GABA(B) receptor blockers, GDPs were evoked by electrical stimulation in the *stratum radiatum* via a theta glass electrode or via a bipolar nickel–chrome electrode. Extracellular and patch-clamp recordings were digitized at 10 kHz with a Digidata 1440A interface card (Axon Instruments) and analyzed offline using MATLAB (MathWorks, Natick, MA, USA) routines.

### Data Analysis

GDPs were detected from the extracellular recordings either from MUA burst analysis as described previously (Khalilov et al., 2015) or from the LFP. In brief, raw data were preprocessed using custom-written functions in MATLAB (MathWorks, Natick, MA, USA). Raw data from extracellular recordings were explored to detect MUA, following which the raw data were down-sampled to 1000 Hz. MUA was detected at a band-passed signal (>400 Hz and <4000 Hz), where all negative events exceeding 3.5 standard deviations were considered to be spikes. Further analysis of extracellular units and LFP data were performed using custom-written, MATLAB-based programs. When MUA bursts were used for the detection and analysis of GDPs, MUA in the CA3 pyramidal cell layer was binned in 20 ms windows and smoothed with a sliding Gaussian window with length of 10 ms. All MUA frequency peaks exceeding 2.5 standard deviations were considered as MUA bursts. MUA bursts were further visually inspected to exclude false bursts caused by artifacts. The times of MUA peaks (Time = 0, hereafter referred to as “GDP time”) were further used for processing of the concomitant patch-clamp recordings through averaging within a −1000 to 3000 ms time window. Statistical analysis was based on Jackknife estimates of the means and standard deviations with the significance level set at *P* < 0.05 assessed using Wilcoxon test. Detection of GDPs from LFP deflections in the pyramidal cell layer that are typically associated with GDPs (see also Ben-Ari et al., 1989) was performed on the lowpass (<5 Hz) filtered LFP trace.

## Results

**Figure 1:**
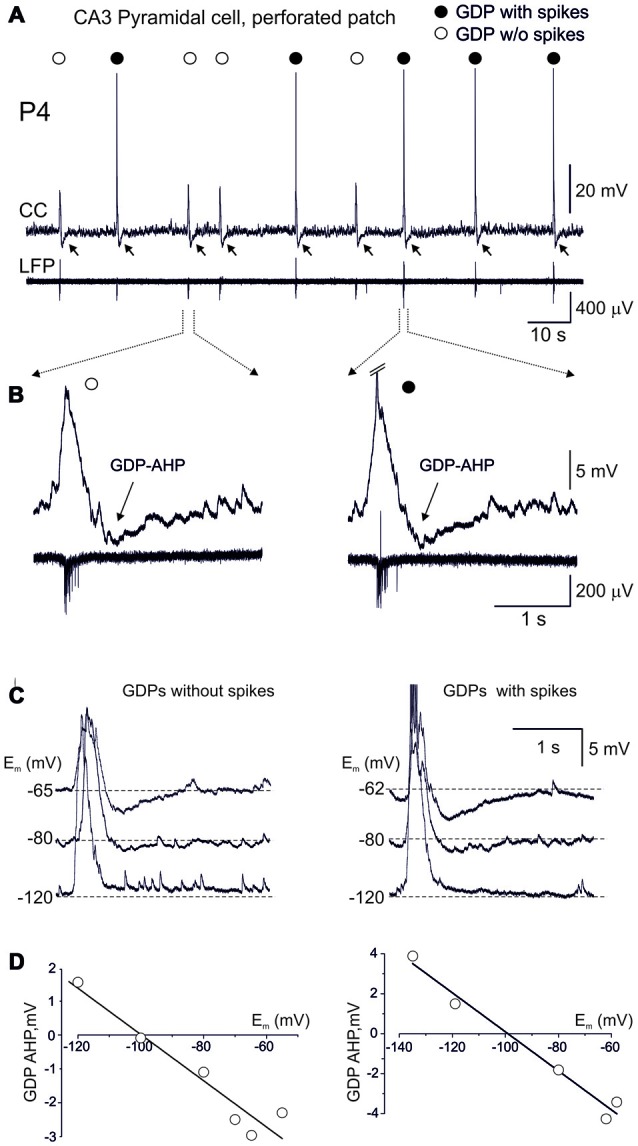
Terminating Giant Depolarizing Potentials (GDPs) afterhyperpolarization (AHP) is expressed in CA3 pyramidal cell independently of whether the cell fires or not during GDP. **(A)** Example traces of gramicidin perforated patch recordings in current-clamp mode (CC) from a CA3 pyramidal cell and local field potential recordings (LFP) from the CA3 pyramidal cell layer. Note that the cell fires (black circles) or not (open circles) during GDPs. Independently of whether cell fires or not during GDPs, GDPs are followed by AHP (GDP-AHP, marked by arrows). **(B)** Examples of GDPs during which the cell remains silent (left) or fires (right, spikes are truncated) during GDPs on expanded time scale. Note that GDP-AHP is expressed independently of whether the cell is activated or not during GDP. **(C)** Example traces of GDPs in current clamp mode at different values of the membrane potential maintained through an injection of the negative current. Left, GDPs without spikes and right, GDPs with spikes. Spikes are truncated. **(D)** Corresponding plots of the GDP-AHP amplitude as a function of the membrane potential. Note that the GDP-AHP reverses at around the potassium reversal potential.

In the present study, we used gramicidin perforated patch-clamp recordings from CA3 pyramidal cells in the neonatal rat hippocampal slices to characterize the properties of the hyperpolarization curtailing GDPs. GDPs were observed as recurrent polysynaptic events associated with MUA bursts and LFP events during extracellular recordings occurring at 0.06 ± 0.01 Hz in all hippocampal slices of P4–8 rats (*n* = 23 cells; Figure 1). The peak in MUA or negative LFP trough during GDPs was taken as a Time = 0 reference point for further analysis of the data obtained using concomitant patch-clamp recordings from individual CA3 pyramidal cells.

### Occurrence of GDP-AHP Does Not Require Firing during GDPs

**Figure 2:**
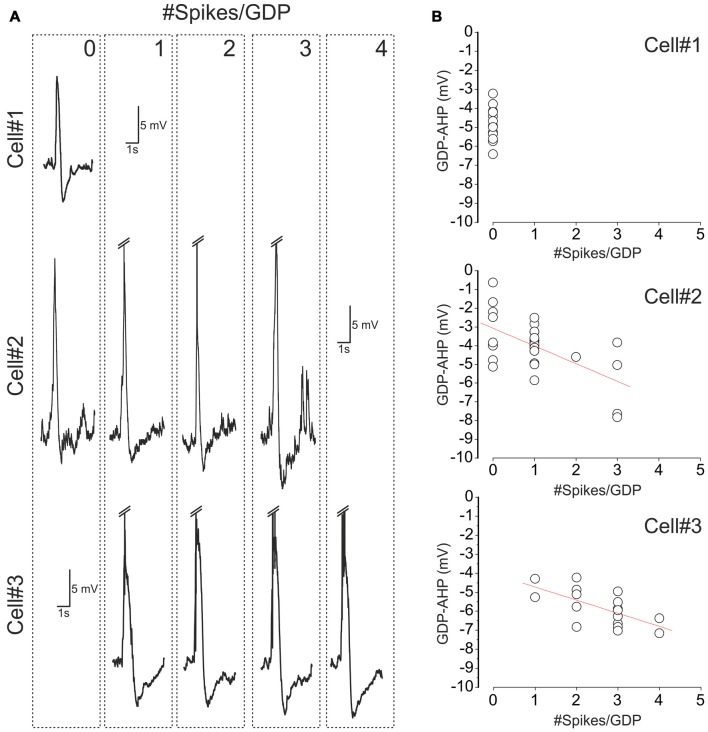
Variability of cell firing and GDP-AHP in CA3 pyramidal cells. **(A)** Examples of GDPs in three CA3 pyramidal cells sorted by the number of spikes that each cell fired during GDPs (the number of spikes per GDP is indicated at the top right corner of the dashed boxes). Spikes are truncated. Note that cell#1 never fired during GDPs, cell#2 fired from 0 to 3 spikes and cell#3 fired from 1 to 4 spikes per GDP. **(B)** Corresponding plots of the amplitude of GDP-AHP as a function of the number of spikes during GDP. Each circle corresponds to an individual GDP. Note that GDP-AHP is present after GDPs without spikes and that the size of GDP-AHP increases with the number of spikes during GDPs.

We analyzed the occurrence of both spikes and GDP-AHP in GDPs, in the hypothesis that GDP-AHP could be generated only as spike rebound. Neurons depolarized during GDPs from the membrane potential of −69.7 ± 2.1 mV (measured at an interval from −1000 ms to −500 ms from the GDPs’ peak) to the peak value of −52.2 ± 2.0 mV (*n* = 23 cells). GDPs were curtailed by AHP attaining peak negative values of −4.7 ± 0.3 mV from the baseline at 712 ± 40 ms after the GDPs’ peak (Figure 1). On average, CA3 pyramidal cells fired 1.9 ± 0.3 APs per GDP (760 GDPs from 23 cells). However, the amount of firing during GDPs was variable in a given cell and between cells. Examples of this variability are shown on Figures 1, 2. In a cell shown on Figures 1A,B, only half of GDPs were associated with spikes whereas the remaining GDPs were only associated with a subthreshold depolarization. Independently of whether the cell fired during GDP or not, all GDPs were curtailed by GDP-AHP. GDP-AHP also occurred after GDPs in cells that never fired during GDPs (Figure 2, cell#1). GDP-AHP following GDPs without spikes attained on average −4.2 ± 0.1 mV (*n* = 163 “silent” GDPs from 13 cells). During injection of the negative holding current, GDP-AHPs reduced in amplitude and reversed in polarity from hyperpolarizing to depolarizing near the potassium reversal potential (*E*_K_ = −99 mV), both during GDPs with and without spikes (Figures 1C,D). Thus, GDP-AHP occurrence does not require cell firing during GDPs and therefore GDP-AHP is generated not only as spike rebound.

### Size of GDP-AHP Increases with Firing Rate during GDPs

**Figure 3:**
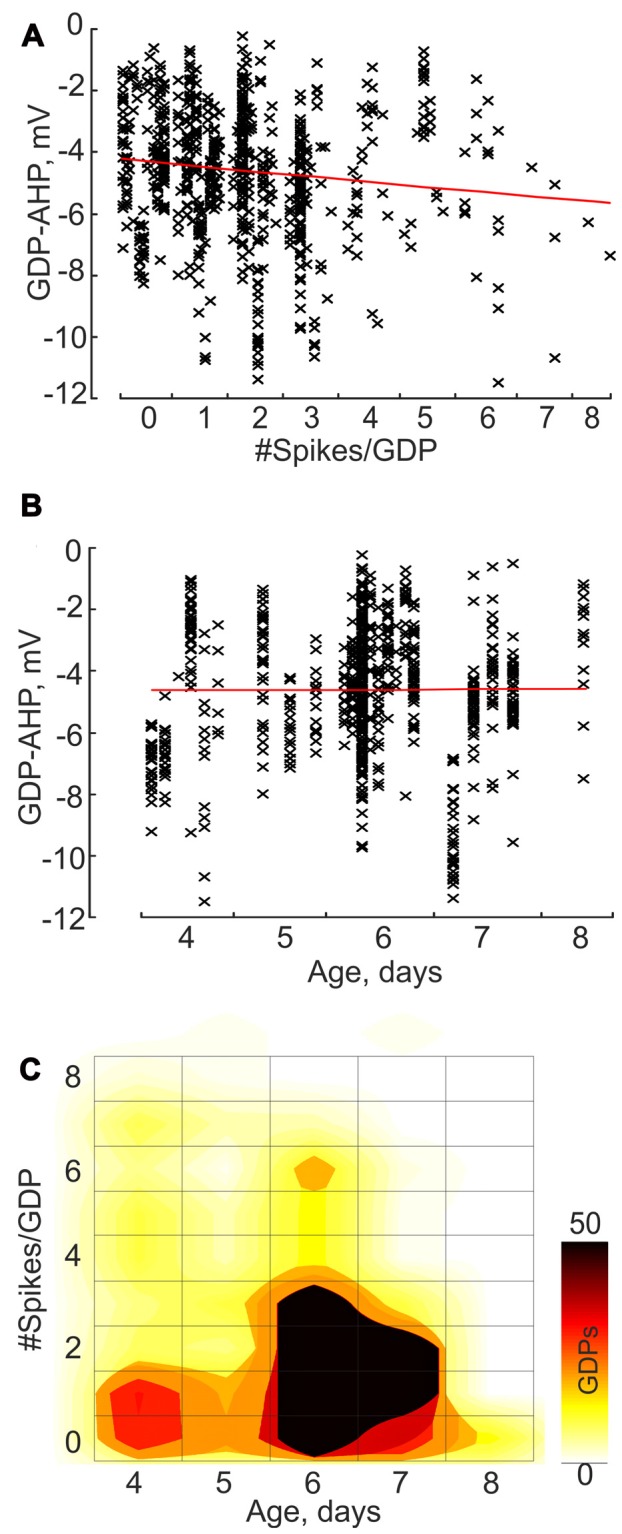
Correlation between the GDP-AHP amplitude, cell firing during GDPs and age: group data. **(A,B)** GDP-AHP amplitude as a function of the number of spikes per GDP **(A)** and the animal age **(B)**. Each data point corresponds to an individual GDP and each column corresponds to an individual cell. Note an increase in the size of GDP-AHP with an increase in cell firing during GDP and no significant change in GDP-AHP size with age. **(C)** Color-coded dependence of cell firing during GDPs on age. **(A–C)** Group data obtained from 23 P4–8 CA3 pyramidal cells.

Next we analyzed how GDP-AHP’ size depends on the firing rate during GDPs. Example recordings of GDPs from three cells with a different amount of firing during GDPs are shown in Figure 2A. Cell#1 never fired during GDPs, cell#2 occasionally fired from 1 to 3 spikes and cell#3 always fired from 1 to 4 spikes during GDPs. In cells #2 and #3, there was a clear correlation between the number of spikes per GDP and the amplitude of GDP-AHP (Figure 2B). We further clustered GDPs from all cells by the number of APs per GDP and found that there is a positive correlation between the size of GDP-AHP and the number of spikes per GDP at the population level (Figure 3A; *r* = 0.118, SD = 2.061; *P* = 0.001, 760 GDPs from 23 cells). Interestingly, within the age group from P4 to P8, GDP-AHP amplitude did not show any significant dependence on age (Figure 3B; *r* = 0.002, SD = 2.076; *P* = 0.95). This is despite of a slight but significant developmental decrease in firing during GDPs (Figure 3C; *r* = −0.166, SD = 1.49; *P* < 0.001; *n* = 760 GDPs from 23 cells). Together, these results indicate that the GDP-AHP contains two components: Component 1, which is independent of cell firing and Component 2, which is positively correlated with the firing of cell during GDPs.

### Potassium Currents Curtail GDPs in Voltage-Clamp Mode

**Figure 4:**
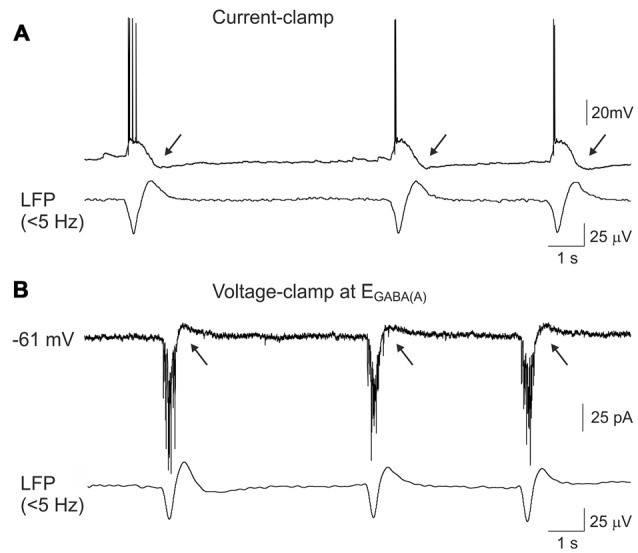
Outwardly directed currents during GDPs-AHP. **(A)** Current-clamp and **(B)** voltage- clamp gramicidin perforated patch recordings of GDPs in a CA3 pyramidal cell and concomintant local field potential (LFP) recordings from CA3 pyramidal cell layer (lowpass filter 5 Hz). Voltage-clamp recordings shown on **(B)** were performed at the holding potential corresponding to the reversal potential of the GABA(A) receptor mediated currents (−61 mV) and the inwardly directed currents during GDPs are mediated by the glutamate receptors. GDP-AHP **(A)** and outwardly directed currents during GDP-AHP under voltage-clamp **(B)** are marked by arrows.

**Figure 5:**
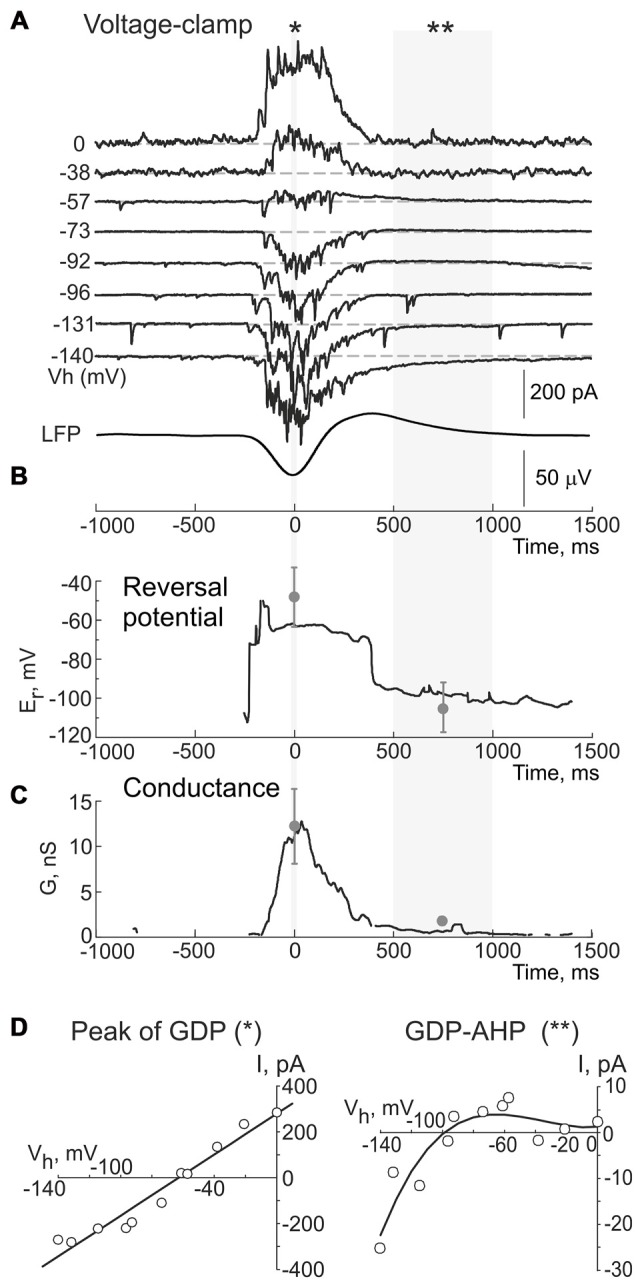
Dynamic changes in the reversal potential of the conductance activated during GDPs and GDP-AHP in a CA3 pyramidal cell. **(A)** Example traces of the currents recorded using gramicidin perforated patch at different holding potentials during GDPs in a CA3 pyramidal cell from a P5 rat. Events are aligned against the troughs of field GDPs (bottom trace). **(B)** Reversal potential and **(C)** conductance of the currents during GDPs in the cell shown in **(A)**. Gray circles and error bars indicate mean ± SD obtained in seven cells. **(D)** Current-voltage relationships of the currents at the peak of GDPs (left, for a period indicated by asterisk in **(A)**, and during the GDP-AHP phase (right, for a period indicated by two asterisks in **(A)**. Note that a reversal potential of the conductance during the GDP-AHP phase is close to the potassium equilibrium and that it displays an inward rectification upon depolarization.

We further attempted to characterize the currents underlying the firing-independent Component 1 of GDP-AHP during voltage-clamp recordings (Figures 4A,B). In voltage-clamp mode, CA3 pyramidal cells displayed bell-shaped barrages of synaptic currents during GDPs. GABA(A) or glutamate receptor mediated postsynaptic currents predominated depending on the holding potential, that is in keeping with the results of previous studies (Ben-Ari et al., 1989; Leinekugel et al., 1997; Bolea et al., 1999; Khalilov et al., 2015). When neurons were recorded near the reversal potential of the GABA(A) receptor-mediated currents (as illustrated on Figure 4B, see also a trace recorded at −57 mV on Figure 5A), the inwardly directed polysynaptic currents during GDPs were composed of a barrage of fast AMPA receptor-mediated EPSCs (see also Bolea et al., 1999; Khalilov et al., 2014, 2015). These EPSC bursts were followed by outwardly directed current occurring during the GDP-AHP phase (indicated by arrows on Figure 4B).

Next, we searched for the origin of the firing-independent Component 1 of GDP-AHP driving force. In this aim, we calculated the instantaneous conductance (*G*) and the reversal potential (*E*_r_) of the transmembrane currents during GDPs from the voltage dependence of the field GDPs triggered current values (Figure 5). Example traces of the currents recorded during GDPs at different holding potentials are shown on Figure 5A; below are shown corresponding changes in *E*_r_ (Figures 5B,D) and *G* values (Figure 5C). We found that at the peak of GDPs, when the maximal *G* values were observed (12.3 ± 4.2 nS, *n* = 6), the *E*_r_ values were of −48.3 ± 15.8 mV (*n* = 6; Figures 5B–D, left plot) and they remained at these levels largely dominated by the GABA(A) receptor mediated chloride conductance (*E*_GABA(A)_ of 63 ± 1 mV; *n* = 6; see also Khalilov et al., 2015) until a transition to post-GDP hyperpolarization. Transition to the GDP-AHP phase was associated with a shift of *E*_r_ to −106 ± 13 mV with the conductance values attaining 2 ± 0.6 nS (*n* = 6; Figures 5A–C). Current-voltage relationships of the conductance activated during the GDP-AHP phase revealed an inward rectification at the potentials more positive than −60 mV (Figure 5D, right plot). Thus, the currents activated during the GDP-AHP phase under voltage-clamp conditions display features of an inwardly rectifying potassium conductance. These results suggest an involvement of the postsynaptic GABA(B) receptor activated inwardly rectifying potassium currents to the firing-independent Component 1 of GDP-AHP.

### Current-Voltage Relationships of the GABA(B) Receptor Activated Currents

**Figure 6:**
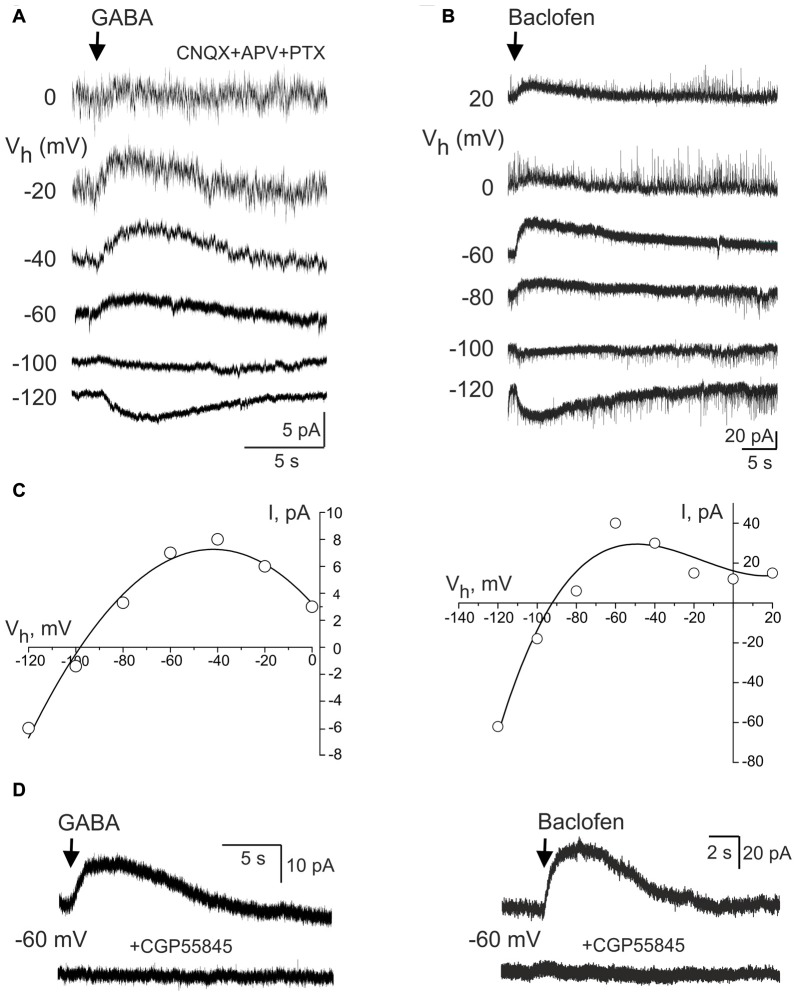
GABA(B) receptor activated currents in CA3 pyramidal cells. **(A,B)** Whole-cell voltage-clamp recordings of the currents evoked by brief local application of GABA in the presence of the glutamate and GABA(A) receptor antagonists **(A)** and GABA(B) receptor agonist baclofen **(B)** at different holding potentials. **(C)** Corresponding current-voltage relationships of the GABA(B) receptor activated currents. Note a reversal near the potassium equilibrium and an inward rectification. **(D)** The effect of the selective GABA(B) receptor antagonist CGP55845 on the responses evoked by GABA (left) and baclofen (right).

We further studied properties of the GABA(B) receptor activated currents in CA3 pyramidal cells during whole-cell recordings to match them with the Component 1 of GDP-AHP. GABA(B) receptor-mediated responses were evoked by a brief local puff application of GABA (100 μM for 100 ms) in the presence of the GABA(A) receptor antagonist picrotoxin (100 μM) and ionotropic glutamate receptor antagonists CNQX (10 μM) and d-APV (50 μM; *n* = 5 cells; Figure 6A). Alternatively, GABA(B) receptors were activated by a brief local puff application of baclofen in the absence of any blockers (300 μM for 100 ms; *n* = 6 cells; Figure 6B). At holding potential of −60 mV, the amplitude of the GABA and baclofen-evoked responses were of 8.6 ± 1.7 pA (*n* = 5) and 47.3 ± 18.0 pA (*n* = 6), respectively. The reversal potential of these responses was of −97 ± 0.1 mV (*n* = 4) and −98 ± 2 mV (*n* = 6), respectively (Figure 6C). Both types of responses were characterized by inward rectification (Figure 6C) and they were completely suppressed by the GABA(B) receptor antagonist CGP 55845 (100 nM) or CGP 54626 (1 μM; Figure 6D). Thus, the responses evoked by the activation of GABA(B) receptors and the currents activated during GDPs-AHP phase under voltage-clamp conditions display similar current-voltage characteristics including the potassium reversal potential and inward rectification.

### Blockade of GABA(B) Receptors Reduces GDPs-AHP

**Figure 7:**
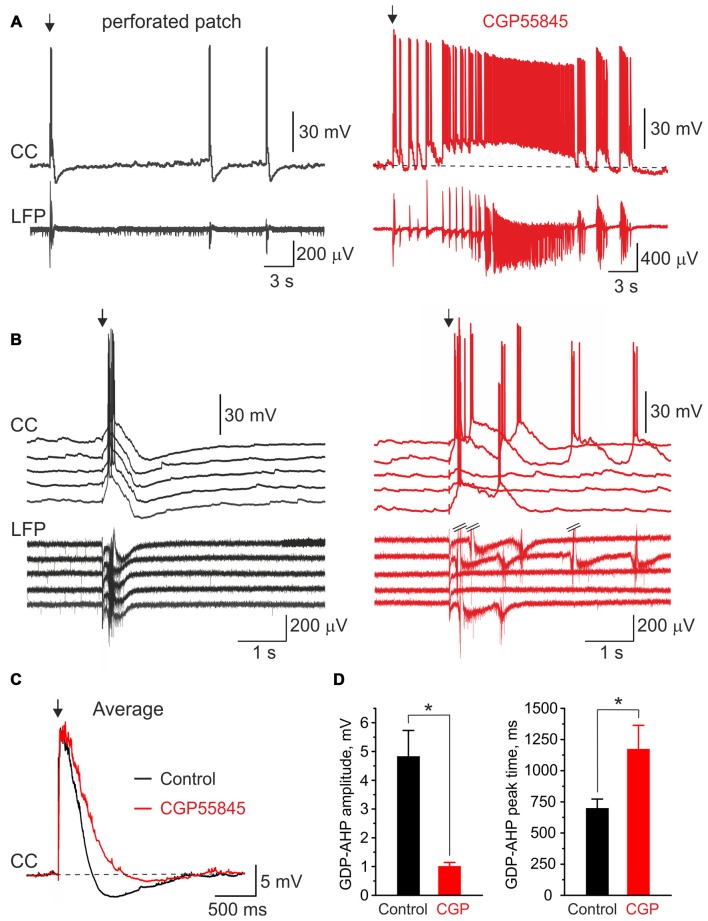
Blockade of GABA(B) receptors suppresses GDP-AHP. **(A)** Gramicidin perforated patch current-clamp recordings from a CA3 pyramidal cell (CC, top trace) and LFP recordings from CA3 pyramidal cell layer from the hippocampal slices of a P7 rat under control conditions (left, black traces) and after bath application of the GABA(B) receptor antagonist CGP55845 (100 nM; right, red traces). The first GDP on each trace is triggered by the electrical stimulus (arrow) in *s. radiatum*. Note an ictal-like discharge triggered by the electrical stimulus in the presence of CGP55845. **(B)** Example traces of five electrical stimulus-triggered eGDPs on expanded time scale before (left) and after (right) addition of CGP55845. **(C)** Average eGDPs from five P4–P7 CA3 pyramidal cells before (black, 144 eGDPs) and after (red, 115 eGDPs) addition of CGP55845. Note an increase in duration of eGDPs and partial suppression of the GDP-AHP by CGP55845. **(D)** Effect of CGP55845 on the GDP-AHP amplitude (left graph) and the delay of the GDP-AHP peak from the stimulus (right graph). Pooled data from six cells (P4–P7 rats), *P < 0.05.

We further tested the effects of the GABA(B) receptor antagonist CGP55845 (100 nM) on GDP-AHP. In keeping with previous findings (McLean et al., 1996), blockade of GABA(B) receptors exerted robust effects on the network activity in the neonatal hippocampus (Figure 7). This included an increase in the duration of field GDPs and associated MUA discharges and an emergence of the ictal-like epileptiform discharges (*n* = 6 slices, P4–7; Figures 7A,B shows example GDPs on expanded time scale under control conditions (black traces), and in the presence of CGP55845 (red traces)). Because the LFP waveform of GDPs and the duration of MUA bursts changed in the presence of CGP55845, we quantified the effects of the GABA(B) antagonists on GDPs evoked by electrical stimulation (eGDPs, failures during the postictal depression were excluded from analysis). CGP55845 induced the following changes in the eGDP parameters: (i) an increase of the half-time from 196 ± 18 to 279 ± 38 ms (*n* = 6 cells; *P* < 0.05); (ii) an increase in the AP firing during eGDPs from 1.8 ± 0.3 to 2.7 ± 0.8 spikes/eGDP (*n* = 6 cells; *P* < 0.05); (iii) a decrease in the eGDP-AHP amplitude from −4.8 ± 0.9 to −1.0 ± 0.1 mV (*n* = 6 cells; *P* < 0.05) and a shift in the eGDP-AHP peak from 701 ± 71 to 1176 ± 188 ms after the stimulus (*n* = 6 cells; *P* < 0.05; Figures 7C,D). These results are consistent with the hypothesis that postsynaptic GABA(B) receptor activated potassium currents contribute to the activity-independent Component 1 of GDP-AHP.

## Discussion

The principal finding of the present study is that the GDP-AHP in neonatal CA3 pyramidal cells is generated through the cooperation of postsynaptic GABA(B) receptor activated potassium currents (Component 1) and spike-dependent, likely calcium activated, potassium currents (Component 2). These observations extend previous hypothesis on the important roles of GABA(B) receptors in controlling network discharges in the neonatal hippocampus by indicating that not only presynaptic, as suggested previously (McLean et al., 1996), but also postsynaptic GABA(B) receptors, as shown here, are involved in the GDPs’ termination.

While previous studies mainly focused on the mechanisms underlying synchronous depolarization and firing of neurons during GDPs in the neonatal hippocampus, relatively few addressed the mechanisms underlying AHP following GDPs. Thus, Sipila et al. (2006) used whole-cell recordings from CA3 pyramidal cells to provide evidence that GDP-AHP involves calcium-activated potassium channels activated by bursts of APs in CA3 pyramidal cells during GDPs. This hypothesis is supported by a transient increase in intracellular calcium which occurs in CA3 pyramidal cells during GDPs, and which can be prevented by voltage clamping neurons to prevent their firing (Leinekugel et al., 1997; Garaschuk et al., 1998). In agreement with this hypothesis, we found that the size of GDP-AHP increases with an amount of neuronal firing during GDPs, with the stronger bursts likely causing larger calcium transients and more robust activation of the calcium-activated potassium channels. Yet, in addition to this activity-dependent Component 2 of GDP-AHP, we also observed activity-independent Component 1 of GDP-AHP. Component 1 was observed following GDPs without cell firing in current-clamp recordings and it also persisted in voltage-clamp recordings during the GDP-AHP phase. This Component 1 had a reversal potential of the potassium conductance and displayed inward rectification similar to that of the GIRK channels activated by the GABA(B) receptors (Gähwiler and Brown, 1985; Sodickson and Bean, 1996; Lüscher et al., 1997). Also, GDP-AHP was sensitive to the GABA(B) receptor antagonist CGP55845.

Previous studies showed that pharmacological suppression of GABA(B) receptors strongly increases GDPs duration and evokes ictal-like epileptiform discharges in the developing hippocampal network (McLean et al., 1996). In keeping with the delayed maturation of postsynaptic GABA(B) receptor activated potassium currents in cortical neurons, and with the early expression of presynaptic GABA(B) receptor mediated inhibition, it has been suggested that the network discharge during GDPs is terminated by activation of presynaptic GABA(B) receptors. Our results indicate that postsynaptic GABA(B) receptor activated potassium conductance is activated and importantly contributes to the hyperpolarization of CA3 pyramidal cells following GDPs thus contributing to the termination of the network discharges during GDPs. Similar roles for pre- and postsynaptic GABA(B) receptors in terminating cortical network excitation have been also evidenced during termination of the GABA(A) receptor antagonists-induced interictal-like bursts in hippocampal slices from fetal non-human primates (Khazipov et al., 2001) and UP-to-DOWN state transitions in slices of medial entorhinal cortex of adult animals (Craig et al., 2013). In the future studies it would be of interest to estimate the relative roles of the pre- and postsynaptic GABA(B) receptors in termination of GDPs using selective pharmacological blockers or genetic suppression of the pre- and postsynaptic GABA(B) receptors. This will also provide sure evidence on the involvement of the postsynaptic GABA(B) receptor activated potassium channels to the GDP-AHP as suggested by results of the present study.

Our findings extend the views on the “dual” roles of GABA in the developing networks. It has been previously shown that the action of GABA, via GABA(A) receptors, dynamically changes during GDPs—from excitatory at the onset of GDPs to inhibitory at the GDPs’ peak, and this dynamic switch, which is controlled by the membrane potential, has been suggested to control the network preventing epileptiform transformations in the neonatal hippocampal network (Khalilov et al., 2015). Here, we show that in addition to this dynamic control of the network excitability through GABA(A) signaling mechanism during the “catharsis” phase of GDPs, GABA also exerts postsynaptic inhibitory actions during the termination phase of GDPs via postsynaptic GABA(B) receptors. This renders GABA with an ability to control the two critical check-points in network dynamics, through the rapid GABA(A) receptor mediated inhibition during the catharsis phase and through the delayed GABA(B) receptors mediated potassium currents underlying the Component 1 of GDP-AHP during the termination phase. During GDPs associated with neuronal firing, this mechanism cooperates with an additional AP-dependent Component 2 of GDP-AHP. Previously, it has been suggested that the AP-dependent GDP-AHP plays an important role in synchronization of the intrinsic bursting of CA3 pyramidal cells during GDPs (Sipila et al., 2006). Activity-independent GABA(B) receptor mediated GDP-AHP may secure such synchronization by setting an internal clock of spontaneous bursting in synchrony with population independently of firing during cycles of slow GDP-oscillations as it also occurs during generation of oscillations in the disinhibited thalamus (von Krosigk et al., 1993).

## Author Contributions

RK conceived the project and designed experiments. IK, MMu, EJ and RK performed the experiments. MMi, IK, MMu and RK analyzed the data. RK wrote the article.

## Conflict of Interest Statement

The authors declare that the research was conducted in the absence of any commercial or financial relationships that could be construed as a potential conflict of interest.
